# Autochthonous human case of *Echinococcus ortleppi* cystic echinococcosis in Brittany, Western part of France

**DOI:** 10.1016/j.fawpar.2025.e00264

**Published:** 2025-04-17

**Authors:** Brice Autier, Marion Baldeyrou, Heithem Jeddou, Coralie Barrera, Jean-Pierre Gangneux

**Affiliations:** a Université de Rennes, CHU Rennes, Inserm, EHESP, Irset (Institut de recherche en santé, environnement et travail) - UMR_S, 1085 Rennes, France; b Service de Maladies Infectieuses et Réanimation Médicale, CHU de Rennes, Rennes, France; c Service de chirurgie hépatobiliaire et digestive, CHU Rennes, Rennes, France; d Department of Parasitology-Mycology, National Reference Centre for Echinococcoses, University Hospital of Besançon, France; e UMR CNRS 6249 Laboratoire Chrono-environnement, Université Bourgogne-Franche-Comté, Besançon, France

**Keywords:** Echinococcosis, *Echinococcus granulosus*, Cestode

## Abstract

Human cystic echinococcosis (CE) is a worldwide infection due to the larval stage of *Echinococcus granulosus sensu lato*, a taeniid tapeworm of canids. Identification of the causative agent at species level relies on molecular methods such as DNA sequencing or species-specific qPCR, which are rarely used for routine case management. Among the different species within the *E. granulosus* complex, *Echinococcus ortleppi* (*E. granulosus* genotype G5 former “cattle strain”) has been reported in only 19 human cases worldwide, including 3 in France. We report the 20th case of *E. ortleppi* cystic echinococcosis, which is an French autochthonous case of a patient without usual risk factors for CE, and living in an area not known to be endemic for *E. ortleppi*. This case highlights that medical community should be aware of the benefits from molecular epidemiology in understanding the landscape of parasitic diseases.

## Highlights

• *Echinococcus ortleppi* is a rare species within the *E. granulosus* species complex.
• Cystic echinococcosis (CE) remains endemic in countries with very low prevalence.
• CE can be acquired in the absence of traditional risk factors.
• Improved clinical, epidemiological and molecular documentation of CE is needed.

## 1 Introduction

Cystic echinococcosis (CE) is a parasitic disease, mainly affecting the liver or the lungs, due to the *Echinococcus granulosus sensu lato* larva called metacestode. In the parasite lifecycle, dogs are infected with the adult stage in their intestines, following ingestion of metacestode containing protoscoleces. Humans and intermediate hosts become infected by ingesting eggs emitted with the infected dog's feces and develop metacestodes in their tissues. A great diversity of mammals can act as intermediate hosts, but the lifecycle of *E. granulosus sensu lato* is considered to be mainly domestic and typically involves livestock animals. Some intermediate hosts are more frequently associated to specific species within the *E. granulosus* complex.

Most human CE cases are asymptomatic for years or even decades (Junghanss et al., 2008). If present, the clinical signs of human CE are generally related to the location and the size of the cyst(s). The main risk of complication is the rupture of the cyst, which can induce severe anaphylaxis and/or secondary dissemination of the parasite. During the natural pathophysiological process, the host immune response can isolate the parasite, leading to its inactivation and calcification. Once calcified, cysts are usually asymptomatic and therefore not investigated nor diagnosed. This explains the difference between the high proportion of inactive cysts in population-based studies (50 % of untreated CE cases) and their relative low frequency in clinical practice (Brunetti and Tamarozzi, 2023). The activity stage of the cyst can be evaluated, although imperfectly, by ultrasound (US) imaging, Magnetic Resonance Imaging (MRI) or Computed-Tomography scanner (CT-scan).

The *E. granulosus* species complex includes *E. granulosus sensu stricto* (G1-G3 genotypes, former “sheep and buffalo strains”), *E. canadensis* (G6/7 former “camel and pig strains” and G8/G10 former “cervid strains”), *E. ortleppi* (G5 former “cattle strain”) and *E. equinus* (G4 former “horse strain”), which accounts for 77 %, 22 %, 1 % and 0 % (2 cases) of reported human CE cases in Europe, respectively (Casulli et al., 2022). Another species in the complex, *E. felidis*, is quite different as it has lions (*Panthera leo*) as definitive hosts, is therefore restricted to Africa, and has not been described yet in human infections. If the *E. granulosus* complex species were defined on the basis of their genetical and epidemiological divergence, it is not known if their involvement in human CE is associated with clinical, diagnostic or therapeutic specificities. This could be related to the scarcity of molecular documentation of human CE cases, whether due to the lack of available tools or the lack of need for case management (Casulli et al., 2022). While *E. granulosus sensu stricto, E. canadensis and E. ortleppi* are known to still be endemic in livestock animals in France (Umhang et al., 2020), autochthonous cases are rarely described (Casulli et al., 2022). Here we describe a case of *E. ortleppi* CE, in a patient with no history of travel outside his native region (Brittany, Western part of France).

## 2 Materials and methods

### 2.1 Data collection and ethics approval

Epidemiological and clinical data were collected from the medical records and patient questionings. Biological data were collected from the routine workup of the laboratory. Especially, the *Echinococcus* serology was performed using the marketed assays *Echinococcus granulosus* ELISA (Bordier Affinity Products, Crissier, Switzerland), Indirect Hemagglutination Assay (IHA, Fumouze Diagnostics, Biosynex, Illkirch-Graffenstaden, France) and Western-Blot (ECHINOCOCCUS Western Blot IgG, LDBio Diagnostics, Lyon, France) following manufacturer's instructions. The patient was informed and his signed consent for the use of his anonymized data was collected. The study was approved by the Ethics Committee of the Rennes University Hospital no 20.133.

### 2.2 Sequencing analysis

Sequencing analysis of the mitochondrial *cox1* gene was performed on biological material obtained from surgical excision of the parasite. After DNA extraction using the EZ1 device (Qiagen, Courtaboeuf, France), a 446 bp partial sequence of the gene was amplified using the JB3 (TTTTTTGGGCATCCTGAGGTTTAT) and JB4.5 (TAAAGAAAGAACATAATGAAAATG) primers (Bowles et al., 1992). The reaction mix was done using the HotStart™ Taq reagents (Qiagen), with 0.6 μM of each primer, 4 mM MgCl_2_, 200 μM dNTP and 3 U of Taq polymerase in a final volume of 25 μL, including 5 μL of DNA extract. The amplification was performed on Veriti™ thermal cycler (Applied Biosystems, Thermo Fisher Scientific, Illkirch-Graffenstaden, France) with an initial denaturation of 15 min at 95 °C, then 45 cycles of 30 s at 95 °C, 40 s at 50 °C and 1 min at 72 °C. After migration in FlashGel™ system (Lonza Bioscience, Colmar, France), PCR product was purified using polyacrylamide gel (Bio-Gel® P-100, Bio-Rad®, Marnes-La-Coquette, France), and the Sanger sequencing reaction was performed with the BigDye Terminator v3.1 kit (Applied Biosystems), purified with the BigDye XTerminator® purification kit (Applied Biosystems), and run on the Applied Biosystems™ 3500 Genetic Analyzer. The forward and reverse sequences were obtained with the Sequence Analysis Software v5.4 (Thermo Fisher Scientific), cleaned and aligned to define the consensus sequence. The obtained 347 bp sequence was then submitted to the BLAST® tool from the National Center for Biotechnology Information (NCBI, https://blast.ncbi.nlm.nih.gov/Blast.cgi) (Sayers et al., 2021) and used for phylogenetic tree construction using *E. granulosus* complex species *cox1* sequences from Genbank (https://www.ncbi.nlm.nih.gov/genbank/). The phylogenetic relationships were determined by the Maximum-Likelihood method with 2,000 bootstrap replicates, using the MEGA11 software (Tamura et al., 2021). The *cox1* sequence of *Echinococcus multilocularis* (AB777916) was used as outgroup.

## 3 Results and discussion

**Fig. 1:**
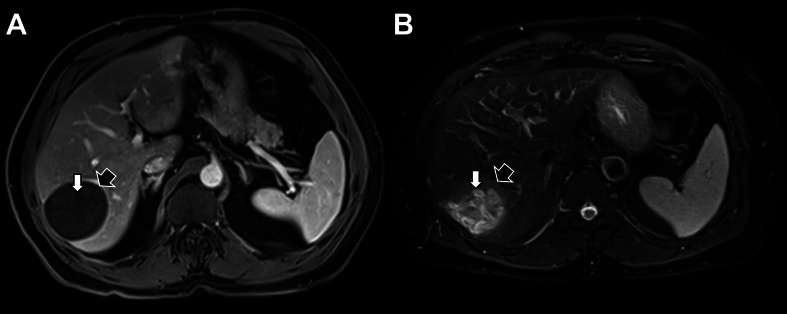
Magnetic Resonance Imaging (MRI) of the *Echinococcus ortleppi* hydatid cyst. Typical 4CE cyst imaging showing a floating membrane (white arrows) in a solid matrix (black arrows), in T1 (A) and T2 (B) acquisition.

The patient was a 65-year old male followed by his pulmonologist for Chronic Obstructive Pulmonary Disease (COPD) treated with inhalated olodaterol and tiotropium. Due to the persistence of symptoms, a chest CT-scan was performed in 2023 September, which incidentally revealed the presence of a liver lesion. A further MRI allowed to identify the lesion as a 6 cm cyst of the VI segment, typical of hydatid cyst (Fig. 1). The cyst was classified CE4, which usually leads to therapeutic abstention, known as the “watch and wait” approach for inactive cysts (Brunetti et al., 2010). However, it has been decided to surgically remove the cyst due to its position on the edge of the liver, making it at increased risk of rupture.

**Fig. 2:**
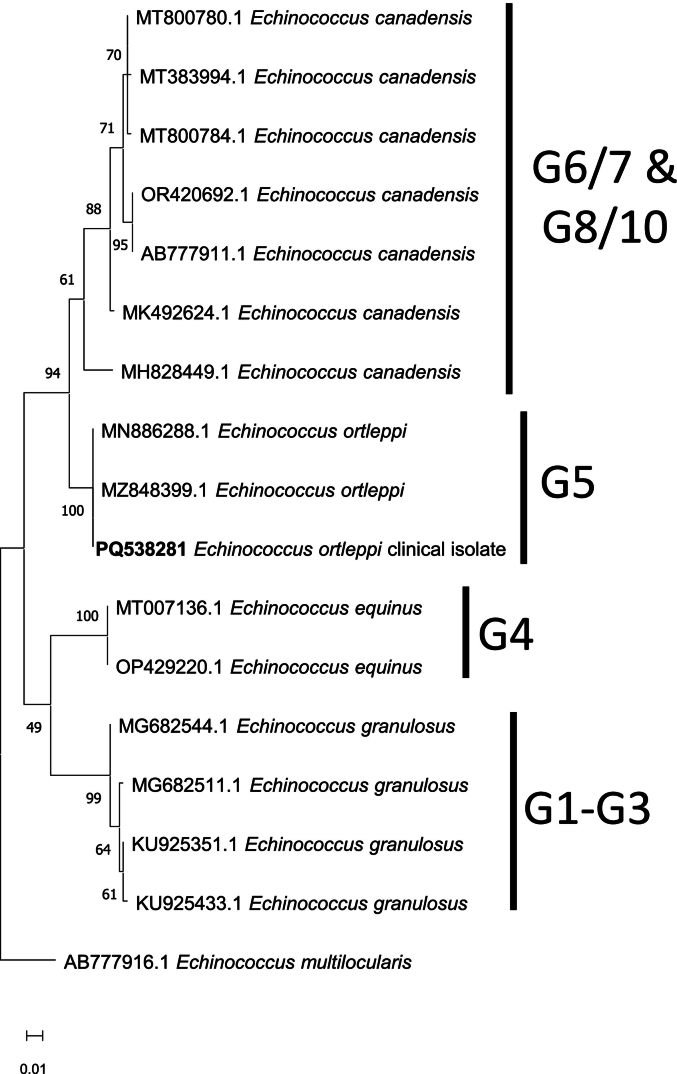
Phylogenetic relationships by the Maximum-Likelihood method between the present *Echinococcus ortleppi* clinical isolate and other species of *Echinococcus*, based on a 347 bp partial sequence of the *cox1* gene. Nodal values are bootstrap values expressed in percentages out of 2000 replicates. *Echinococcus multilocularis* (AB777916) was used as outgroup. The newly generated sequence is shown in bold.

Usual blood tests (complete blood count, liver enzymes, ionogram) were normal at the time of diagnosis. Echinococcosis serology was negative by ELISA and IHA, however Western Blot from LDBio Diagnostics, which has been described to be more sensitive than ELISA and IHA (Vola et al., 2019), was positive with an *Echinococcus granulosus* profile (7 kDa band). The parasite was surgically removed by partial hepatectomy with albendazole prophylaxis (400 mg/day, adapted dosage following therapeutic drug monitoring) in 2024 April. The partial *cox1* gene sequencing allowed to identify the causative agent as *Echinococcus ortleppi* (Fig. 2), with 100 % identity with reference sequences from Genbank. Pathological examination confirmed the parasitic nature of the partially calcified cyst, without visible protoscolex. MRI follow-up at 2 months post-surgery was satisfying and the patient did not complain from any clinical sign.

Including this case, *E. ortleppi* has been reported in 20 human cases of CE worldwide, 7 in Europe, 9 in Asia, 3 in Americas (South America and Mexico) and 1 in South Africa (Yoshida et al., 2024). The cysts were mainly located in the liver (10/20, 50 %), but also in lungs (4/20, 20 %) and other organs (heart, spleen, spine). The 4 French cases of *E. ortleppi* CE were not restricted to an area, as they have been reported in Brittany, Vendée, Burgundy and Jura regions (Grenouillet et al., 2014; Basmaciyan et al., 2018). These 4 regions form a belt spreading from −1.5° to 6° of longitude, and restricted to 47–48° of latitude. Cattle is the most common livestock animal in these areas, as it is for most of the French territory. Of note, these human cases were not described in areas where livestock animals infected with *E. ortleppi* were detected in a 2012 national survey (Umhang et al., 2020). It remains possible, however, that cattle infected with *E. ortleppi* has been living close to these patients, without being identified.

Originally described as a cattle strain, this species is present and maintained in Europe due to cattle breeding. For this reason, it is actually considered that infection with this parasite is directly related to cattle. However, the patient from the present case was not a farmer (he worked as a fisherman), had lived his entire life in the city of Fougères, Brittany, France (around 20.000 inhabitants), and had never traveled outside the region. He had 2 dogs in his life, one during his childhood and a second which died in 2014. If the chronology is compatible with an infection due to this latter dog, this one had not clear access to cattle carcasses thus to *E. ortleppi* cysts. Altogether, no clear source of infection has been identified for this patient: putatively, his last dog could have been infected with adult stage of *E. ortleppi*, or the patient could have been exposed to *E. ortleppi* eggs in fresh food products (*e.g.* vegetables, berries) (Barosi and Umhang, 2024). The occurrence of human infections with *E. ortleppi* in France highlights that CE is a worldwide disease and can be acquired even in countries with very low prevalence. Also, this suggest that incidence is probably underestimated as many cases, such as the present one, are asymptomatic. Furthermore, it emphasizes the need for increased efforts in molecular epidemiology of helminths, especially agents of echinococcosis, to improve our understanding of the parasitic diseases landscape.

## CRediT authorship contribution statement

**Brice Autier:** Writing – original draft, Visualization, Methodology, Investigation, Formal analysis, Data curation, Conceptualization. **Marion Baldeyrou:** Writing – review & editing. **Heithem Jeddou:** Writing – review & editing. **Coralie Barrera:** Writing – review & editing. **Jean-Pierre Gangneux:** Writing – review & editing, Validation, Supervision.

## Declaration of Competing Interest

The authors declare that they have no known competing financial interests or personal relationships that could have appeared to influence the work reported in this paper.
